# Cystic Teratoma Resection With Ovarian Preservation in a Pediatric Transgender Male: A Case Report

**DOI:** 10.7759/cureus.29161

**Published:** 2022-09-14

**Authors:** Angelo Federico, Mehana Patel, Kristina Cummings

**Affiliations:** 1 Medicine, Lake Erie College of Osteopathic Medicine, Bradenton, USA; 2 Psychiatry, Lake Erie College of Osteopathic Medicine, Elmira, USA

**Keywords:** transgender care, ovarian torsion, ovarian cystectomy, gender-affirming care, cystic teratoma

## Abstract

Ovarian teratomas are germ cell-derived tumors that are classically reported in the literature to occur in cisgender female patients. While this is statistically the most common patient population that they are diagnosed in, they can also occur in transgender men with ovaries who have not undergone a previous oophorectomy. Because of the lack of research and literature regarding this unique patient population, decisions regarding the treatment of these neoplasms are controversial. Here, we report the case of a pediatric transgender male who developed an ovarian teratoma and discuss specific considerations for treating ovarian neoplasms in the transgender population.

## Introduction

Transgender individuals have a gender identity that differs from the sex that they were assigned at birth; the number of people who identify as transgender in the United States is estimated to be almost 1 million [1]. This unique patient population is subject to numerous health disparities and limited access to health care relative to cisgender people. The gender-affirming care that may be sought to treat one’s gender dysphoria may range from medical management, such as hormone therapy and puberty blockers, to more invasive interventions, such as surgical chest and genital reconstruction [2]. There is no one-size-fits-all approach to gender-affirming care as patients may pursue the treatments that they feel are best tailored to suit their individualized medical needs. Androgen treatment is used in female-to-male patients for the elimination of female secondary sex characteristics and for the induction of male secondary sex characteristics [3]. While a female-to-male transition is a clinical indication for long-term testosterone administration, the knowledge of the effects that androgen treatment has on female reproductive organs is scarce [4]. While the World Professional Association for Transgender Health considers both hysterectomy and bilateral salpingo-oophorectomy medically necessary gender-affirming procedures for transgender males, not all patients choose to undergo these procedures [5]. Due to the presence of ovaries, transgender men who have not undergone an oophorectomy are still at risk of developing ovarian neoplasms. This case report discusses the occurrence of an ovarian cystic teratoma that developed in a transgender male.

## Case presentation

**Figure 1:**
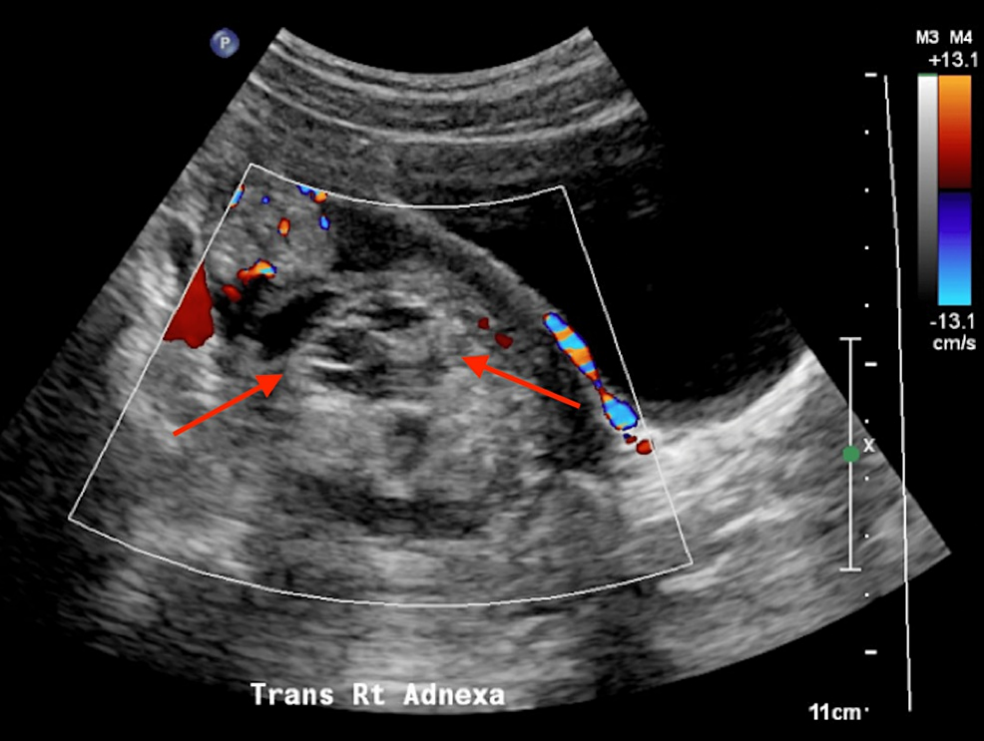
Transverse view of the pelvic ultrasound showing the heterogeneously echogenic region (red arrows) within the right adnexa without internal vascular flow.

**Figure 2:**
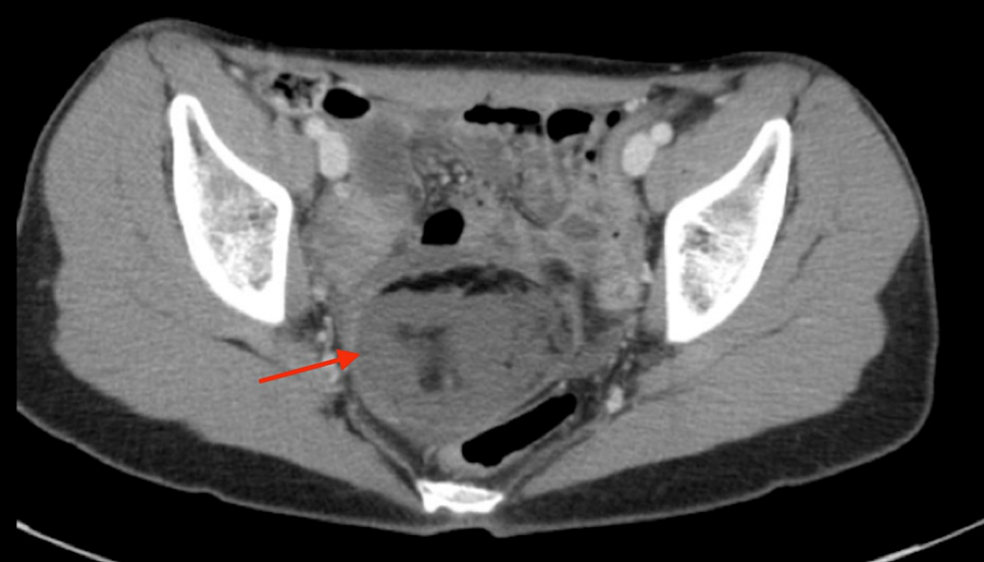
Axial view of the CT abdomen/pelvis showing the complex mass (red arrow). CT: computed tomography

**Figure 3:**
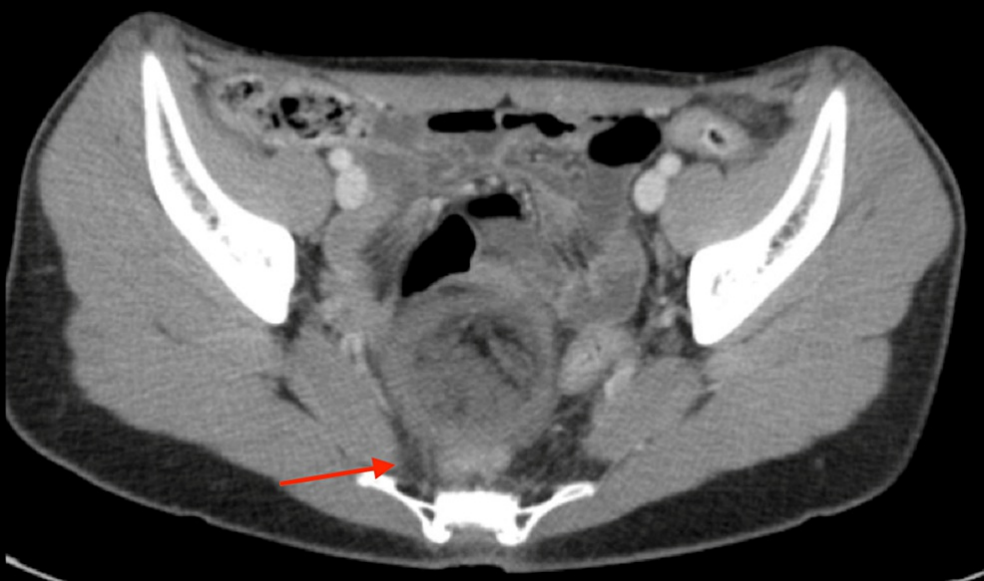
Axial view of the CT abdomen/pelvis showing distended loops of the bowel with compression of the bowel within the sigmoid colon (red arrow). CT: computed tomography

A 16-year-old Caucasian transgender male with a history of longstanding gender dysphoria, depression, and anxiety presented to the emergency room with right lower quadrant pain for two days. The patient had no prior surgical history. Current medications included amitriptyline, Risperdal, and testosterone cypionate. The patient previously used leuprolide acetate to help achieve amenorrhea. On physical examination, the abdomen was tender to palpation in the right lower quadrant. Laboratory evaluation was within normal limits. Abdominal and gallbladder ultrasounds were negative for pertinent findings. Pelvic ultrasound demonstrated a heterogeneously echogenic region measuring 5.4 × 5.1 × 5.0 cm within the right adnexa without internal vascular flow (Figure 1). Ovarian torsion could not be excluded as the right ovary was not discretely identified on ultrasound. Computed tomography (CT) of the abdomen and pelvis demonstrated a complex mass measuring 6.5 × 5.5 × 4.8 cm with multiple densities within the pelvis, likely representing a mature teratoma (Figure 2). There was no evidence of torsion on CT as the ovaries were enhanced appropriately. CT also showed distended loops of the bowel with a transition point at the sigmoid colon adjacent to the pelvic mass (Figure 3).

Due to concern for mature teratoma, an outpatient follow-up to schedule surgery was recommended. After counseling regarding the benefits and risks of surgery, patient goals, and future plans for gender-affirming surgeries, the patient elected to undergo Da Vinci robotic-assisted laparoscopic right ovarian cystectomy via mini-laparotomy rather than unilateral oophorectomy to allow for maximum ovarian tissue preservation.

**Figure 4:**
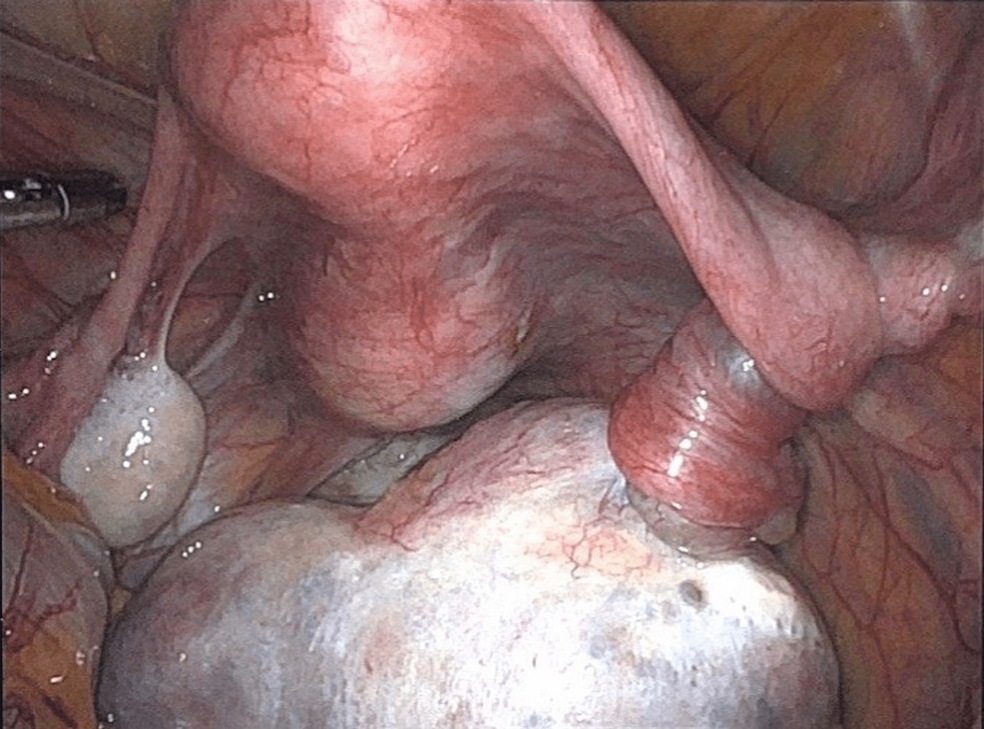
Anterior view of cystic teratoma and the resultant right adnexal torsion.

Intraoperative examination under anesthesia demonstrated a fullness of the posterior cul-de-sac upon palpation, which was confirmed via laparoscopy to be the right ovarian cystic teratoma. This had resulted in torsion of the right ovary approximately five times (Figure 4). The right adnexa was untwisted and observed for approximately 10-15 minutes to allow the venous congestion to dissipate and to allow for proper revascularization of the tissue. A specimen bag was used during the cystectomy to collect the dermoid, which was completed successfully without rupture. The specimen bag was then removed via mini-laparotomy upon completion of the procedure. The patient tolerated the operation well and was discharged with instructions to follow up in seven weeks.

**Figure 5:**
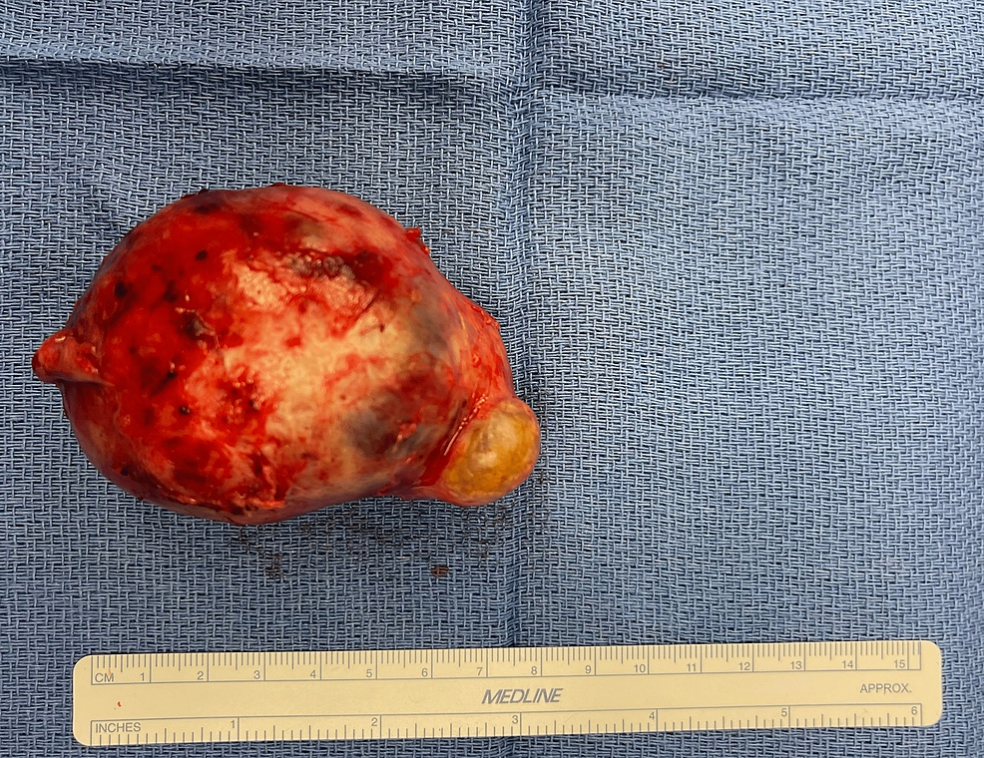
Specimen after removal via mini-laparotomy measuring 8.0 × 5.5 × 5.0 cm.

The surgical pathology report for the specimen was described grossly as a right ovarian cystic teratoma with an irregular fragment of membranous soft tissue measuring 8.0 × 5.5 × 5.0 cm (Figure 5). The specimen was sectioned to show a well-circumscribed cavity measuring 4.5 cm in diameter filled with a yellow-tan gelatinous material and hair. Also identified within the cavity was a calcified portion of tissue measuring 1.0 × 0.3 × 0.3 cm.

## Discussion

Transgender individuals face substantial barriers to accessing health care, including healthcare professional bias, lack of general knowledge about best practices, and limited accessibility to gender-affirming surgery [6]. Providers with less exposure to the transgender population have reported lower levels of comfort with discussions regarding gender identity and counseling for required medical care [7]. As there is difficulty in determining the prevalence of reproductive cancers in the transgender population, current guidelines suggest routine screening of retained internal organs. Consideration of the health risks posed for patients based on their age and individual anatomy helps create a structured and personalized approach to their care [8].

The patient in this case had already started his medical transition via exogenous testosterone therapy. Desired masculine changes induced by testosterone therapy include changes in body fat redistribution, increase in muscle mass and strength, and menstrual suppression [9]. The role of testosterone treatment in ovarian tumor progression is uncertain. Prior clinical studies investigating patients diagnosed with polycystic ovarian syndrome who are exposed to higher levels of endogenous androgens have failed to show any correlation to ovarian cancer progression [10]. However, these studies may not be applicable to the use of exogenous testosterone therapy to achieve cisgender male baseline testosterone levels, especially in a transgender male for masculinization.

Because the patient was diagnosed with a cystic teratoma complicated by ovarian torsion, complete resection of the teratoma was required. Ovarian teratomas, which are germ cell-derived tumors, are the most common type of ovarian neoplasm diagnosed in pediatric patients [11]. While much is known about ovarian tumors, less is known regarding their malignant potential and the possible use of ovarian-sparing operative techniques for definitive treatment. Therefore, risk stratification must be used to determine the appropriate treatment. Many obstetrician-gynecologists cite the fear of leaving behind a potential malignancy as a reason to perform an oophorectomy [12]. While surgical resection has historically been the standard treatment for ovarian teratomas, there are still potential risks associated. Due to the resultant decrease of both estrogen levels and ovarian follicular reserve, unilateral oophorectomy may lead to impaired fertility [13]. Preservation of ovarian tissue and the production of estrogen also provides reproductive benefits maintaining follicular development for future oocyte cryopreservation. The decrease in estrogen also causes concern regarding future gender-affirming surgery.

When considering surgical treatment for ovarian neoplasms, special consideration is given to the patient’s age and future fertility goals, along with the size of the neoplasm. The desire for future gender-affirming surgery may need to be considered as well. Endogenous estrogen plays a significant role in future masculinizing surgery as it helps preserve growth, functionality, and blood flow to select reproductive structures. These structures, such as the vagina, vulva, and clitoris, are used in certain masculinizing surgeries. Metoidioplasty, a specific masculinization surgery, utilizes the tissue of the clitoris and neighboring skin to create a neophallus [6]. If desired, a scrotoplasty can also be performed using the rotational flaps of the labia majora to place the created scrotum into a proper anatomical position [6]. Insufficient development or atrophy of these structures in an adolescent transgender male can serve as a disadvantage in the consideration of candidacy for gender-affirming surgery. Given the importance of gender-affirming surgery, which has been shown to reduce suicide risk, the risks and benefits of oophorectomy versus cystectomy were imperative to consider for this patient [14]. Because a strong desire for future reassignment surgeries was expressed, in this case, the consideration of long-term patient goals was crucial when determining the mode of resection. Ultimately, cystectomy with ovarian preservation was recommended rather than the standard treatment.

**Figure 6:**
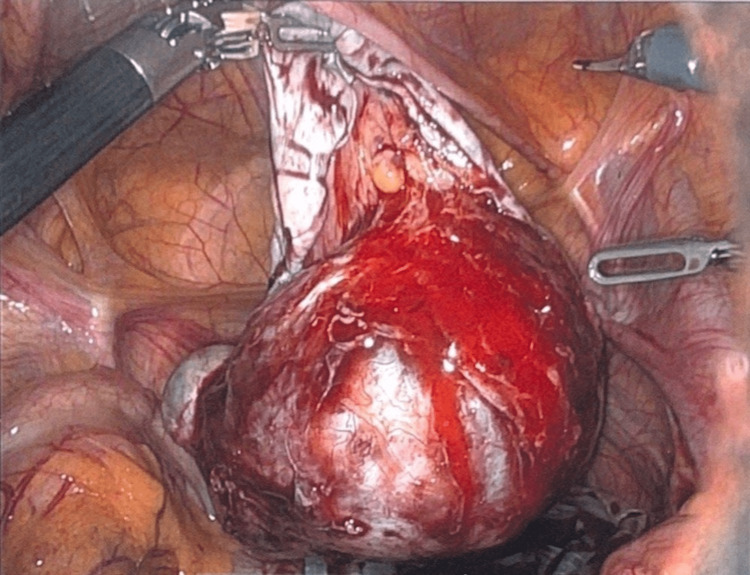
Right cystic teratoma mid-cystectomy with ovarian tissue preservation.

Further studies regarding the prevalence and management of ovarian tumors in transmasculine individuals are required. Varying surgical approaches to the excision and maximization of tissue preservation have led to controversies in the surgical management of such patients. In this case, the patient elected to undergo a Da Vinci robotic-assisted laparoscopic right ovarian cystectomy via mini-laparotomy (Figure 6). There is a common consensus that an operative laparoscopy is an ideal method of choice for removing an ovarian cystic teratoma because it offers the advantages of a shorter hospital stay, less intraoperative blood loss, reduced postoperative pain, fewer postoperative adhesions, and better cosmetic results [15]. Utilization of the Da Vinci surgical system may improve the precision of the dissection; however, employing the use of such a system for the resection of ovarian tumors in the adolescent population is not widely studied. More in-depth research on the approach is needed to make further advancements in the procedures and techniques utilized in treating these unique cases.

## Conclusions

With an increasing number of patients undergoing medical transitions each year, healthcare providers need to be prepared to adequately screen, educate, and treat these patients to provide appropriate quality of care. Concerns such as the effects of exogenous testosterone use on the risk of developing ovarian neoplasms, endogenous effects of estrogen on future gender-affirming surgery, and potential fertility considerations are just a few topics that require more attention and research. Without such knowledge, it can become difficult to thoroughly discuss the questions these patients may be struggling with regarding their future health, creating a barrier when attempting to maximize the quality of care. Due to such limited information on transgender health, researching the risks and benefits of such procedures, as well as their physiologic and psychological effects, can help further enhance care and minimize the barriers faced by these patients when accessing health care.
